# A 3D map of the islet routes throughout the healthy human pancreas

**DOI:** 10.1038/srep14634

**Published:** 2015-09-29

**Authors:** Constantin Ionescu-Tirgoviste, Paul A. Gagniuc, Elvira Gubceac, Liliana Mardare, Irinel Popescu, Simona Dima, Manuella Militaru

**Affiliations:** 1 National Institute of Diabetes, Nutrition and Metabolic Diseases “N.C. Paulescu”, Bucharest, Romania; 2 National Institute of Pathology “Victor Babes”, Romania; 3 Faculty of Engineering in Foreign Languages, Politehnica University of Bucharest, Romania; 4 Institute of Genetics, University of Bucharest, Bucharest, Romania; 5 University of Agronomic Sciences and Veterinary Medicine, Bucharest, Romania; 6 Clinical Institute of “Fundeni”, Bucharest, Romania

## Abstract

Islets of Langerhans are fundamental in understanding diabetes. A healthy human pancreas from a donor has been used to asses various islet parameters and their three-dimensional distribution. Here we show that islets are spread gradually from the head up to the tail section of the pancreas in the form of contracted or dilated islet routes. We also report a particular anatomical structure, namely the cluster of islets. Our observations revealed a total of 11 islet clusters which comprise of small islets that surround large blood vessels. Additional observations in the peripancreatic adipose tissue have shown lymphoid-like nodes and blood vessels captured in a local inflammatory process. Our observations are based on regional slice maps of the pancreas, comprising of 5,423 islets. We also devised an index of sphericity which briefly indicates various islet shapes that are dominant throughout the pancreas.

Islets of Langerhans are directly responsible for maintaining homeostasis. Both their size and shape determine the uniqueness of these micro-organs. The three-dimensional relationship between islets is crucial in orchestrating the metabolic adjustment. However, this relationship is unknown. One of the major drawbacks in etiopathogenesis knowledge of different types of diabetes is largely represented by an excessive extrapolation of experimental data obtained in several mouse models^1^,^2^,^3^. It turned out that this extrapolation was in many respects a disadvantage when the first immuno-therapeutic approaches to type 1 diabetes (T1D) in humans was initiated^4^. The presence of two glands in the same organ, one with an internal secretion and the other with an external secretion, is more an exception than a rule. While the exocrine pancreas has a regular lobular organization, the endocrine pancreas proves to be uniquely heterogeneous, whether we refer to the total number of islets or to their size. In previous studies, the total number of islets varied between 3.6 and 14.8 million, and the total islet volume has been reported between 0.5 to 1.3 cm^3^ 5,6,7,8. Furthermore, the cellular composition of the islets makes it difficult to accurately calculate the total number of β cells. Detailed studies made on the human islets report a proportion of ~60% for β cells and ~30% for α cells, the remainder being divided between δ cells (<10% - somatostatin-secreting), γ cells (<5% - secreting pancreatic polypeptide), and ε cells (ghrelin secreting)^9^,^10^,^11^. Moreover, human, monkey and mouse islets showed functional differences that correlated with structural differences^9^. It stands to reason that this whole dynamic cellular arrangement inside the islet performs complex functional relationships, otherwise difficult to be determined *in vivo* and *in situ*. If pancreatic β cells are post-mitotic cells in adulthood as proven by some data^12^,^13^,^14^,^15^, the existence of some minimal replications becomes pertinent. Thus, such a minimal replication capability might be explained partially by some young pancreatic β cells appeared through neogenesis or a cell transdifferentiation of commited endocrine cells^16^. This controversial subject regarding β cell replication is reminiscent of numerous studies in various animals, especially done on mice^17^,^18^. Some drug classes tested in animals have maintained this unconfirmed hypothesis that β-cell regenerative potential may be encountered also in humans. This assumption is not unreasonable. For instance, this regenerative potential is routinely encountered in the intestinal epithelium or in hepatocytes. It is possible that in the case of β-cell apoptosis similar processes may be triggered in humans also but it is unlikely that adult pancreatic β cells maintain this replication property. Instead, the neogenesis of β cells may be stimulated in some ductal cells or in cells belonging to the endocrine pathway, stimulating them through different transcription factors to adopt the β cell phenotype^19^. Even in this situation, it is unknown whether these cells can mature and become insulin-producing entities with proper mature secretory vesicles^20^. It is known that type 2 diabetes (T2D) has an increased incidence in older ages, therefore people with a genetic predisposition for this phenotype may inherit the defect of several molecules involved in cell cycle regulation^21^. The current nebula in the pathogenesis of T1D is due to limited access of researchers to study the human pancreas obtained in particular from young diabetic patients. Even so, the pancreas obtained after clinical onset of diabetes expresses overlapping changes that occurred over several years or decades^22^,^23^. Consequently, crucial information is lost on the first changes that occur in pancreatic islets when the first appearance of islet lesions begin to be the main characteristic of this phenotype of diabetes. Identification of this phase could be possible when imaging methods would be sensitive enough to identify such changes^24^,^25^. However, understanding the first “diabetogenic movements” can not be done without a good knowledge of the anatomical pancreatic islet physiology and their relationships with other pancreatic structures in normal individuals. With a good quality pancreas from an organ donor, in the present work we have proposed a detailed analysis of the fundamental characteristics of the pancreatic islet, such as their size, shape and their 3D distribution. The first objective was to quantify the area and perimeter of pancreatic islet regions: head, neck, body and tail. A second objective intended to establish a correlation between the area and perimeter of the islets from four sections of the pancreas in order to identify the preferred islet shapes in each section. A third objective was focused on capturing the dynamics of pancreatic islet distribution using heat maps. Nonetheless, a fourth objective was to compile the four distributions in one general distribution to elucidate the islet density along the pancreas.

## Results

**Figure 1:**
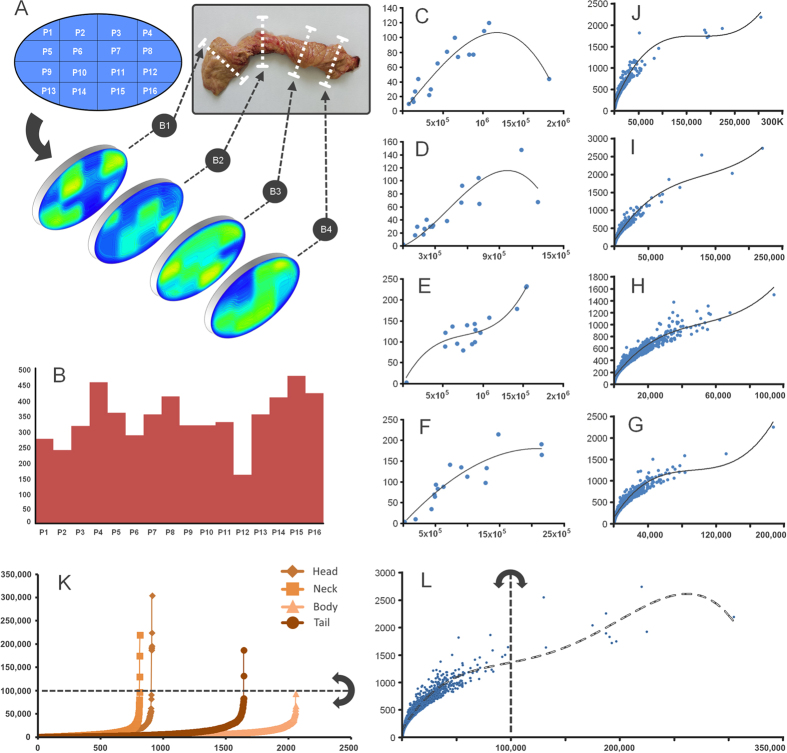
Distribution of pancreatic islets. (**A**) a two-dimensional distribution of pancreatic islets on each section (B1–B4) of the pancreas. The pancreas from the donor and the organizational scheme of the slices are also presented. Dorsal pancreas is represented by Bx[P1-P4] slides whereas the ventral pancreas is represented by Bx[P13–P16] slides. (**B**) the proportion of islets along the four sections. The pancreatic islet distribution on each histological slide depending on the islet number and their average area (μm^2^), it is shown for the: (**C**) head area (B1), (**D**) isthmus area (B2), (**E**) body area (B3), (**F**) tail area (B4). The mean surface area (μm^2^) of the islets is represented on the X-axis whereas the number of islets is represented on the Y-axis. The islet number and their average area per slice (B1–B4) it is shown for the: (**J**) head area (B1), (**I**) isthmus area (B2), (**G**) body area (B3), (**H**) tail area (B4). The trend lines in panels **C** to **H** use a polynomial of order 3. (**K**) distribution of islets ordered by size. For each slice (B1–4), the number of islets is represented on the X-axis whereas the surface area (μm^2^) of the islets is represented on the Y-axis. The diamond shaped points represent islets in the head area. The square shaped points represent islets in the neck area and the points with triangular shape represent islets in the body area. The circular shaped points represent islets in the tail area. (**L**) a global distribution of 5423 islets depending on their perimeter and area. The dotted trend line of panel **L** uses a polynomial order 4. The straight dotted line and the arrows in semicircle in panel **K** and **L**, represent a barrier over which the clusters of islets have been observed.

In this study a healthy human pancreas from a donor has been used to assess the islet characteristics and their spatial distribution. Initially, the first measured parameters consisted of length (**25 cm**) and volume (**45 cm**^**3**^). Onward, four regions of the pancreas were taken into consideration, namely the head (denoted as B1), neck (B2), body (B3) and the tail (B4). From each section of the pancreas we have used one representative cross-sectional slice which has been divided into 16 histological slides. Semi-automatic morphometric measurements were made on each slide in order to detect the specific features of the pancreatic islets. We have analyzed a total of **5423 islets** belonging to the four sections of interest, namely the head (910 islets), neck (813 islets), body (2057 islets) and tail (1643 islets) of the pancreas (Table 1). In Table 1 the number of islets found is shown for each histological slide (B1[P1–P16]–B4[P1–P16]). The maximum number of islets found on a slide has been encountered in the body slice of the pancreas (233 islets), namely on B3[P5] slide, whereas the minimum number of islets was found in the neck slice on the B2[P4] slide. From a longitudinal perspective of the pancreas slices (the total islets found throughout the sections on B1–4[Px] slides), the maximum number of islets has been found on B1–4[P15] slides (473 islets) and the minimum number of islets on B1–4[P12] slides with a total of 156 islets (Table 1). The analysis of morphometric measurements have showed the total **2D area of the islets** on each slice (Table 2). Thus, the 16 histological slides of the head slice (B1) showed a **total islet area** of **9.252** **mm**^2^ and a mean islet area of 0.578 mm^2^ (±0.467), while the neck slice (B2) showed a **total islet area** of **6.960 mm**^**2**^ and a mean islet area of 0.435 mm^2^ (±0.359). The body and tail slices show the largest surface coverage (Table 2). The 16 histological slides of the body slice (B3) showed a total islet area (and a mean islet area) of **14.004 mm**^**2**^ (mean 0.875 mm^2^ ±0.391), which have been similar to the total islet area found in the tail slice (B4), namely **14.186 mm**^**2**^ (mean 0.887 mm^2^ ±0.633). Interestingly, although the two sections (B3 and B4) show similar values for the total islet area, they show a different distribution of the islets on the surface of the two slices (Fig. 1A). From a 2D perspective, the **total islet area** on all (**64 slides**) histological slides show a value of approximately 44.4 mm^2^ (44,401,127.94 μm^2^) with a mean area of 2.775 mm^2^ (±0.467) and a mean of the average islet area on slide of 0.694 mm^2^ ±0.201 (Table 2). A detailed comparison of the islet area and the islet perimeter shows the relative differences in proportions between the two morphometric parameters (Supplementary Table S1 online). Also, a numerical distribution was made between the mean area and the number of islets for each microscope slide (Supplementary Table S2 online). In the next phase we have been interested in determining a series of parameters, namely the islet diameter, the mean islet volume, the total number of islets inside the pancreas, the total volume occupied by islets inside the pancreas and the total 3D surface of islets.

### Islet diameter

To calculate the ***average value of the islet diameter***, two methods have been used due to various shapes of the observed islets, which create a relative disproportionality between their area and their perimeter (ie. a star-shaped islet will have a disproportionate perimeter from the surface area, while a round islet will show a proportionality between perimeter and surface area). The first method took into account the average perimeter (*p*_*m*_) of the islets measured on the histological slide and the mean islet diameter was calculated (1). Thus, the mean islet diameter (diameter of islets using the perimeter - *d*_*ip*_) of the whole pancreas was found to have a value of 113.83 μm (±7.05). The second method used to calculate the diameter of the pancreatic islets has taken into consideration their measured surface area (*a*_*m*_) from the histological slide and the mean islet diameter was calculated using the formula (2). Thus, with this second version of the formula the mean islet diameter (diameter of islets using the area - *d*_*ia*_) of the whole pancreas was found to have a value of 104.02 μm (±5.67). By making an average of the two results, we have calculated the average diameter (mean diameter - *d*_*m*_) between the two values (3), thought to be closer to the actual average value of the islet diameter. The radius (mean islet radius - *r*_*i*_) was also determined (4). Therefore, the mean islet diameter (*d*_*m*_) between the two estimation methods was **108.92 μm** (±6.27) with a radius (*r*_*i*_) of 54.46 μm (±3.13).

### Islet volume

For a full 3D perspective, the ***mean islet volume*** (*V*_*p*_) was calculated according to the average diameter of islets (*d*_*m*_) using the classical formula for the volume (5). Thus, the ***mean islet volume*** (*V*_*p*_) showed a value of 0.00068 mm^3^ (686,994.29 ± 107,297.82 μm^3^) or 0.00069 μl (±0.00011). The mean islet 3D surface area (*A*_*im*_) showed a value of 0.037 mm^2^ (37,464.69 ± 4,109.69 μm^2^) and it was calculated using the formula (6).

### The total number of islets and their volume inside the pancreas

The approximation of the ***total number of islets*** and that of the volume that they occupy, has been made through virtualization. We considered a virtual model in which the pancreas was divided in slices with a thickness equal to the average regional diameter of an islet (*d*_*i*_). We further considered that each virtual slice incorporates the number of islets of the corresponding region. Thus, the number of islets from each virtual slice was summed in order to elucidate the total number of islets in the pancreas (7). Hence, the number of islets in the pancreas was approximated to 3,204,588 islets (**~3.2 million islets**). Inferred by the same rule as above, ***the total islet volume***(8) showed a value of **2 cm**^3^, averaging between the four sections (B1–4) at 0.5 ± 0.1 cm^3^ (Table 3). ***Total islet 3D surface area*** was estimated at **1132 cm**^**2**^ (or 0.1 m^2^), averaging between the four sections at 283 ± 93 cm^2^ (Table 3). Finally, our estimate for the ***islet percentage from the pancreas total volume*** (45 cm^3^) showed an approximate value of **4.487%**. The average islet percentage between sections (B1–B4) showed a value of 1.12 ± 0.3% (see Table 3).

### Islet distribution

**Figure 2:**
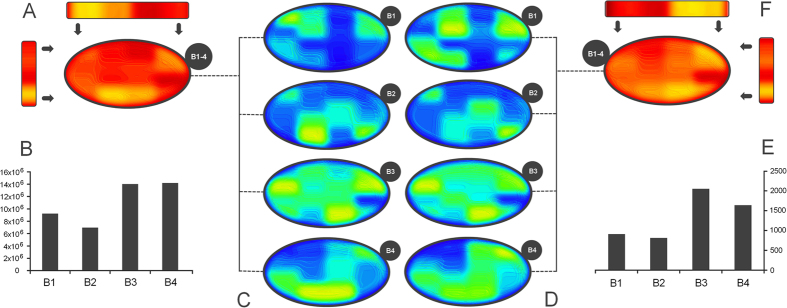
Heat map distribution of the pancreatic islets by their number and surface area. (**A**) Global heat map representing the overlapped distributions of panel **C** (sum of all densities from B1 to B4). The yellow areas represent higher densities of islets while the red color represents areas of a lower density, (**B**) total islet area by slice (B1–4), (**C**) heat map which indicates the surface area occupied by islets, (**D**) heat map representing the islet density in sections B1–B4. The yellow areas represent higher densities of islets while the blue color represents areas of a lower density, (**E**) the total number of islets in each section, namely B1–B4, (**F**) global heat map representing the overlapped distributions from panel **D** (sum of all surface areas from B1–4). The yellow areas represent a higher surface occupancy by islets while the red color indicates a lower surface occupancy.

Distribution of islets inside the pancreas has been one of the main objectives of this study. Using heat maps for an overview of the pancreatic tissue structure, we managed to follow the more dense or less dense parts of the slices (Fig. 1A and Fig. 2D). We also used heat maps to detect the surface areas occupied by islets on each slide (Fig. 2C). By comparing the 2D distributions of the four slices, the first major observation was that islets are grouped in the head of the pancreas. These **islet groups gradually disperse throughout the pancreas** up to the tail region (Figs 1A and 2C,D). Notice that starting from the head up to the tail of the pancreas the dark blue (low-density) area decreases in favor of the light blue area (higher density - Fig. 2C,D). The proportion of islets along the pancreas has been also of interest (Fig. 1B). A particular observation in this case indicates that the average number of islets on B1–4[P12] was very small compared to the average number of islets along other B1–4[Px] slides. Equally interesting, the average number of islets present along B1–4[P4] and B1–4[P15] slides showed the largest values. Moreover, both a distribution of the number of islets and the average islet area/slide and a distribution of the area and perimeter of individual islets has been performed for each slice (Fig. 1C–G). Thus, a clustering of the distribution seen in Fig. 1E indicates an almost uniform scattering of the islets in the body of the pancreas. A global heat map representing the overlapped distributions of the four slices further shows the total number of islets (Fig. 2F) and the sum of all islet areas (Fig. 2A) on B1–4[Px]. Both distributions viewed from the lateral side show a similar pattern. However, when seen from above both distributions show different patterns. If the lateral pattern shows a linear relationship between the total number of islets and the sum of all islet areas, the top view shows a larger islet area on the left side of the slices and a larger number of islets on the right side of the slices (Fig. 2A,F).

### Preferences on islet size

**Figure 3:**
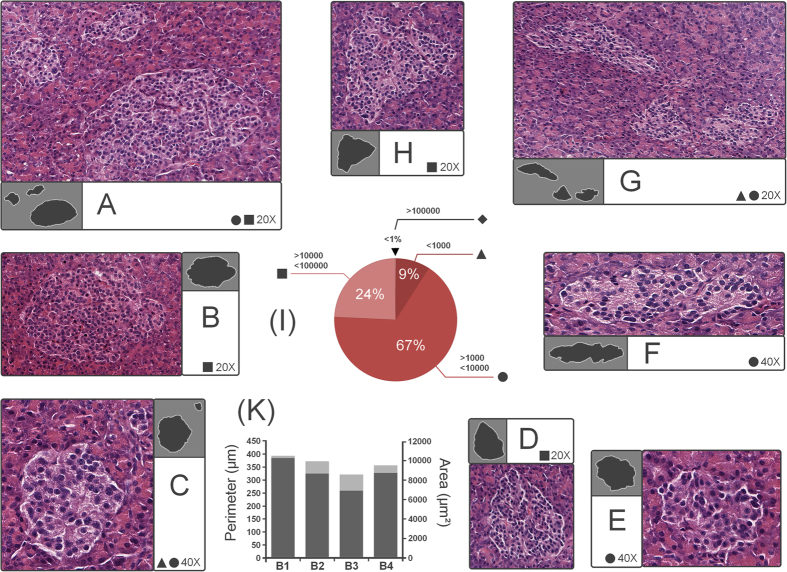
Islet proportions in the human pancreas. (**A**) group of islets (top islet −6356 μm^2^, bottom islet −54,291 μm^2^, left islet −6700 μm^2^) included in the category 1000–10,000 μm^2^ and 10,000–100,000 μm^2^ (field of view 20X), (**B**) islet example (41,708 μm^2^) included in the category of 10,000 μm^2^–100,000 μm^2^ (field of view 20X), (**C**) sample of islets (top islet −386 μm^2^, bottom islet −8814 μm^2^) included in the category <1000 μm^2^ and 1000–10,000 μm^2^ (field of view 40X), (**D**) islet (20,368 μm^2^) belonging in the catgory 10,000–100,000 μm^2^ (field of view 20X), (**E**) example of islet (6282 μm^2^) included in the category 1000–10,000 μm^2^ (field of view 40X), (**F**) islet sample (8805 μm^2^) included in the category 10,000–100,000 μm^2^ (field of view 40X), (**G**) group of islets (top-left islet −14,930 μm^2^, bottom islet −136 μm^2^, bottom-right islet −8402 μm^2^) ranging from 1000-10,000 μm^2^ to 10,000–100,000 μm^2^ (field of view 20X), (**H**) example of islet (29,975 μm^2^) included in the category 10,000–100,000 μm^2^ (field of view 20X), (**I**) islet percentage by size, (**K**) mean islet area (dark-gray on right axis) and mean islet perimeter (light-gray on left axis) by slice (B1–4).

According to the evolutionary parameters of each species, the pancreas structure indicates an optimal size of its inner components, whether endocrine or exocrine. Our data suggests that the human pancreas seems to “prefer” certain dimensions when it comes to islets (Table 4). With a representation of 67% (3614 islets) are those islets with surface areas between 1,000 and 10,000 μm^2^ (Fig. 3A,C,E,G,I and Table 4). This majority is followed with a proportion of 24% (1305 islets) by islets with an area between 10,000 and 100,000 μm^2^ (Fig. 3A,B,D,F–I). Interestingly but not surprisingly, only 9% (493 islets) of the islets have an area up to 1000 μm^2^ (Fig. 3C). Finally, less than 1% are represented by individual clusters of islets (Fig. 3I).

### Islet shapes

The islet shape confers an unique cellular arrangement^9^. Recent data suggests that many changes in islet structure and function associated with diabetes are attributable to hyperglycaemia alone and are reversed when blood glucose is normalized^26^. Thus, the uniqueness of this heterogeneous conformation has a deep functional implication. Pancreatic islets have various shapes and are generally difficult to define in this regard. In our case two major questions were considered: 1) how close are the islets of Langerhans to a perfect spherical structure in diferent regions of the human pancreas ? 2) if the pancreatic islets are too far from a perfect spherical structure then how irregular in shape are these islets? With regard to the first question we have devised an index of sphericity (*I*_*s*_) by considering two islet parameters (9). An absolute value of the islet perimeter (*P*_*m*_) measured on the bidimensional histological slides and a relative parameter, namely the mean islet diameter (*d*_*m*_). However, as a first reference point, we have used the ratio between the circle perimeter (*Cp*) and diameter (*Cd*), namely π (7). Next, the same steps have been applied for the ratio (*I*_*π*_) between the mean islet perimeter and the mean islet diameter (8). The distance from the ideal proportions were quantified through the index of sphericity (*I*_*s*_), in this case as a ratio between *π* and *I*_*π*_ (9). Thus, we have roughly measured how much an islet shape deviates from the ideal shape of a circle (and consequently from an ideal sphere). The islet mean perimeter and diameter were taken into account for each slice (B1–4) of the pancreas, and the distances from the ideal proportions of the circle (π) were evaluated (Table 5). Thus, the islets with irregular shapes showed a disproportion between perimeter and diameter (*I*_*s*_ tends to move away from 1 to higher positive values), while islets with round shapes approached the perimeter and diameter proportions of a circle (*I*_*s*_ tends more towards 1). The islets from the pancreas tail show a ratio (*I*_*π*_ = 3.2526) closer to π, suggesting that in average the islets from the pancreas tail are closer to the ideal spherical structure compared to other sections (*I*_*s*_ = 1.0353). This method also suggests that islets located in the neck of the pancreas exhibit proportions (*I*_*π*_ = 3.3268) that are the most distant from the ideal (π) and exhibit various irregular islet shapes (*I*_*s*_ = 1.0589).

### Islet clusters

**Figure 4:**
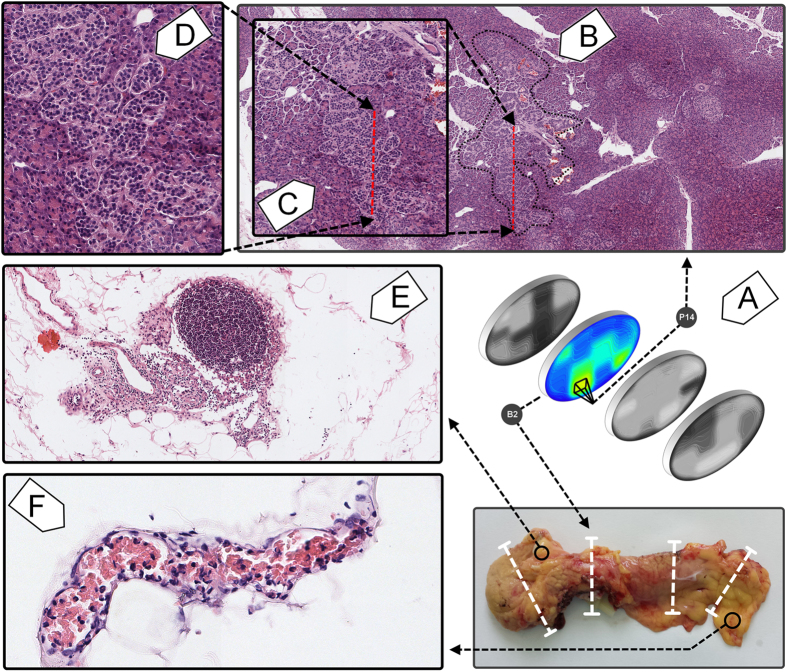
Islet clusters and the status quo inflammatory process of the peripancreatic adipose tissue. (**A**) relative location of the islet cluster on B2(P14) slide (neck of the pancreas). The panel also shows the relative location of the neck of the pancreas and the heat map derived from it, which further indicates the surface area occupied by islets. (**B**) islet cluster boundaries (black dotted line) on B2(P14) slide (field of view 4x), (**C**) window in to the lower part of the islet cluster (red dotted line), (**D**) close view of the islets inside the cluster (field of view 20x), (**E**) lymphoid organized tissue inside the peripancreatic adipose tissue, taken from the head slice of the pancreas (field of view 10x). The reddish stain on the left represents a processing error. (**F**) inflammatory process captured on a blood vessel taken from the peripancreatic adipose tissue of the tail slice of the pancreas (field of view 20x). The blood vessel is filled with red blood cells and inflammatory cells (neutrophils, lymphocytes, eosinophils).

With regard to our study, perhaps one of the most interesting observations has been the detection of islet clusters. The islet distribution by surface area seems to indicate a “threshold” size above 100,000 μm^2^ (Fig. 1K,L). It appears that larger islets, that exceed an area of 100,000 μm^2^ are typically clusters of islets (Fig. 4A–D). From our observations, the structures that are above these dimensions represent clusters of islets. Double-blind morphometric measurements have revealed a total of 11 islet clusters on all four slices (Fig. 1K,L). Six clusters have been detected in the head slice (B1) of the pancreas, three in the neck slice (B2) and two in the tail slice (B3). Islet cluster-like structures were absent in the body slice (B4) of the pancreas (Fig. 1K,L). Moreover, these islet clusters seem to prefer the periphery of the pancreas. A relevant example of an islet cluster is found in the neck of the pancreas and it is represented by B2[P14] slide where the acinar tissue and blood vessels are intercalated among islet groups (Fig. 4B,D). On a closer inspection, the encapsulation boundaries can be observed between islets located in the cluster (Fig. 4D). Given the known heterogeneity of this organ, the question that we have asked ourselves was if these clusters are routinely a part of healthy human pancreas (in individuals without a familial predisposition for T1D or T2D) or they are just a feature only representative for this particular pancreas. In the future we intend to study this organ from other human donors to see if these structures are regularly found in the healthy human pancreas. Also, the islet clusters may be crucial for islet transplantation procedures. Our data shows that islet clusters comprise of small islets that gather tightly along larger blood vessels (Fig. 4B,D). Small human islets are less vulnerable to hypoxia and comprise of more β-cells with higher insulin content than large islets^27^,^28^. Although the body and tail of the pancreas contain many small solitary islets that can be easily isolated, when transplanted into the portal vein of the liver, they lack of intimate connections with the main bloodstream. On the other hand, our data shows that islet clusters appear with a higher frequency in the head and the neck of the pancreas (Fig. 1K). Thus, islet clusters contain larger blood vessels which might be directly connected through microsurgical methods to the patient’s bloodstream circulation (Fig. 4B,D). Perhaps their microsurgical isolation would also be problematic considering that these anatomical structures may be particularly rare and hard to find *in vivo*. Nevertheless, the heterogeneity of the pancreas is perhaps only exceeded by the peripancreatic adipose tissue, where other observations were made in this case. Inside the pancreas the immune responses were absent. In the peripancreatic adipose tissue these immune responses were captured in blood vessels (Fig. 4F). Moreover, many different lymphoid-like structures have been observed in the peripancreatic adipose tissue (Fig. 4E). Thus, it seems that the inflammatory process in the peripancreatic tissue could be a constant, dynamic and common process surrounding the healthy human pancreas. These two observations lead us to believe that the peripancreatic adipose tissue is heavily involved into the initiation mechanisms of both T1D and T2D (with respect to the genetic predisposition for one or the other phenotype). These observations lead us to wonder if perhaps the immune response on beta cells is in fact initiated through induction from the peripancreatic tissue.

## Discussion

The three dimensional distribution of islets in the healthy human pancreas has rarely been studied in detail, largely due to restrictions related to medical ethics or due to an apparent lack of the islet organization extrapolated from previous studies^29^. The formation of islets is determined during embryonic life through the intervention of multiple transcription factors^12^,^30^. This process continues several years after birth^15^,^31^,^32^,^33^,^34^. During this period, numerous islet modifications are pertaining the normal state of the young pancreas, such as β cell or α cell replication outbursts, followed by adjustments of their number through apoptosis^15^,^35^,^36^,^37^. Nevertheless, it is unknown how this adjustment process ultimately determines the ratio inside the pancreatic islet between β cells, α cells, γ cells, δ cells and ε cells^38^. It is possible that the final ratio inside the pancreatic islet may be the result of ad-hoc intercellular “negotiations”, constrained by time-dependent environmental factors.

### The 2 grams of metabolic brain

However, a series of studies have been made on the cell type ratio inside the human islet^9^,^11^,^39^. Overall, the isolated human islets contain β cells in a proportion of 57,1%, α cells in a proportion of 32.6%, and δ cells in a proportion of 10.2% (Table 6). From a total islet volume of **2.02 cm**^3^, β-cells show a volume of **1.15 cm**^**3**^ (around 2.6% from the pancreas volume), α-cells show a volume of **0.66 cm**^**3**^ (around 1.5% from the pancreas volume) and δ-cells show a volume of **0.21 cm**^**3**^ (nearly 0.5% from the pancreas volume). Furthermore, to approximate the total mass of different endocrine cell types, the specific density (1.08 g/cm^3^) of the pancreas tissue has been used^40^,^41^. Thus, we evaluated the total mass of the islets at **2.2 g** (13). Accordingly, we have also evaluated the total mass of β-cells at roughly **1.25 g**, α-cells at **0.7 g** and δ-cells at **0.22 g** (Table 6). Based on our donor’s global parameters, we have approximated an islet volume of **27 mm**^3^**/kg** of body mass (around **42,728 islets/kg** of body mass). Thus, by extrapolating the specific densities of tissues, nearly **0.03 g** of isles regulate the homeostasis of **1 kg** of tissue. Also, following the same assumption of an uniform density in all tissue types, about **0.016 g** of β-cells regulate the homeostasis of **1 kg** of tissue.

### The 3D routes of the pancreatic islets

**Figure 5:**
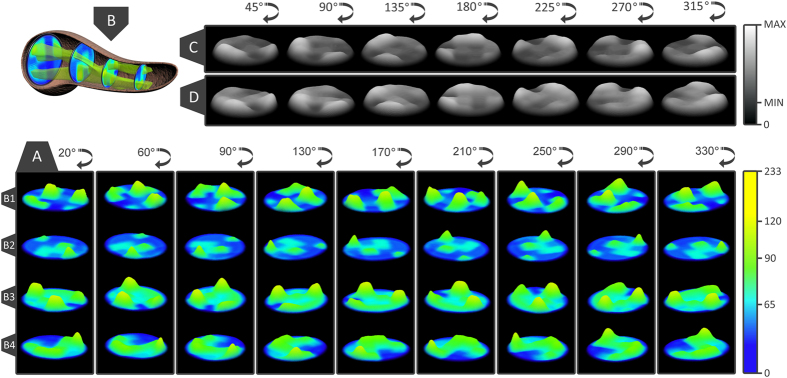
A three dimensional distribution of islets within the human pancreas. (**A**) rotational view of the islet landscapes on sectons B1–4, representing the density of islets. Yellow areas represent the maximum density of islets (233 islets) and blue areas represent the minimum density of islets. (**B**) the 3D islet route through the human pancreas, from the head section to the tail section of the pancreas, (**C**) a rotational view of the global islet distribution (all B1–4 slices combined), representing the density of islets through the human pancreas, (**D**) a rotational view of the surface areas occupied by islets through the human pancreas. Light gray areas represent the maximum density/surface area of islets from all B1–4 slices and dark gray areas represent the minimum density/surface area of islets from all B1–4 slices.

**Figure 6:**
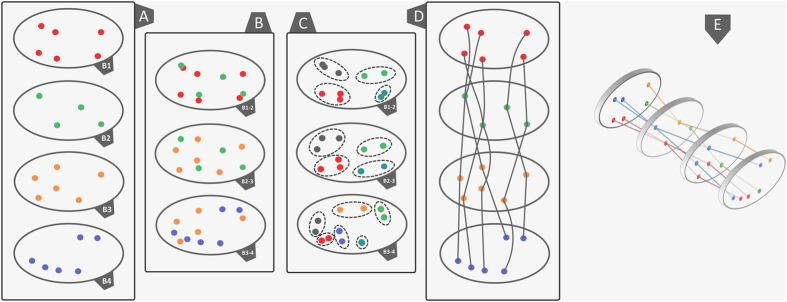
The 3D routes of human pancreatic islets. (**A**) the highest density peaks recorded from the superposition of eight heat map distributions, namely the B1–4 distribution of islets by number and the B1–4 distribution of islets by surface area. Depending on the slice, a distinct color has been assigned to each set of data points. (**B**) an overlay of the B1-B2, B2-B3 and B3-B4 metric spaces, (**C**) clusters indicating the closest data points between neighboring slices. The colors change their meaning to accommodate an identity to each cluster. (**D**) a 2D evaluation of the islet routes according to the clusters shown in panel **C**. (**F**) a 3D evaluation of the islet routes. In order to provide an identity to each islet route, the colors change their meaning once again.

Most often it is considered that the islets are scattered in the human pancreas without an apparent structure^27^. Our model supports a three-dimensional organization of the islets (Fig. 5B). We have observed several high density scattering routes (Fig. 6E). A 3D view of the 2D heat map distributions have indicated the highest islet density peaks (bright yellow - Fig. 5A). Thus, the connections between slices have been determined by using the k-means clustering method (although a Bayesian approach can also represent a good alternative). In order to apply the k-means clustering method (14), the highest density peaks (Fig. 2C,D - a peak threshold associated with bright green) of the four slices (B1–4) have been overlapped. The distance between density peaks counterparts of neighboring slices has been evaluated (Fig. 5A). Thus, in our model the nearest density peaks (closest data points) of two adjacent slices have been connected and considered an islet route (Fig. 5B). Initially, a number of peaks have been recorded on each slice, namely 6 density peaks on B1, B3-4 and 4 density peaks on B2 slice (Fig. 6A). An overlay of metric spaces indicated the number of clusters by evaluating the shortest distance between data points of two adjacent slices (Fig. 6B,C). By overlapping the head-neck data points and the neck-body data points we have noticed the presence of four clusters on each superposition, whereas the body-tail superposition has shown a total of six clusters (Fig. 6C). A 2D evaluation of the islet routes has been made by connecting data points within each cluster (Fig. 6D). Different islet densities on a slice dictate the relative shape of an islet route due to differences between start and stop surfaces of the bidimensional plane of the slices (Fig. 5B). The straight lines intend only to show the densest areas that correspond from slice to slice, according to the k-means clustering method (Fig. 6E). Thus, the 3D model took into account both the calculated routes and the surface areas of the most dense regions on the slices. Alternatively, we also show the islet routes as straight lines for the ease of understanding the connection between slices.

The three-dimensional representation of islet routes has allowed a better assessment of their spatial characteristics (Fig. 6E and Supplementary Figure S3 online). Thus, on the right side of the slices two islet routes were identified (Fig. 6E - green and orange lines). With slight deviations, these islet routes retain a constant position along the pancreas. On the other hand, on the left side of the slices there are four islet routes that converge or diverge from slice to slice (Fig. 6E - red and blue lines). For instance, the islet routes converge from the head to the neck section and diverge from the neck to the body and tail sections. Also, an interesting observation is that between the neck and tail section two islet routes keep their divergence in an almost linear manner (Fig. 6E - blue lines). Further, from B1 slice to B2 slice the islet routes exchange their number in favor of size (Fig. 2C,D). Starting from B2 to B3 slice, the islet routes exchange the islet size in favor of the islet number (Fig. 2C,D).

### Thoughts for the future on the expansion of unknowns

A continuation of these studies is an impetuous for the entire structure of the human pancreas. In the near future we wish to continue this approach by initiating a new study which takes into consideration the relationship of the islets with the blood vessels and ducts. Overall, this study begins to address a significant gap in our anatomical understanding of human islets in relation to the pancreas as a whole. Prior histological studies of the human pancreas have taken into consideration mainly small tissue regions. Most often these regions were rarely specified, and if specified then no exact location was provided.

While to our knowledge no previous study has investigated the distribution of islets within the human pancreas, some studies have been conducted on the mouse pancreas^42^,^43^,^44^. Furthermore, additional research would be practical for a better assessment of the islet clusters with regard to the distribution of individual islet sizes and the composition of endocrine cell types. In light of the above observations additional questions of interest also include: 1) given that these islet clusters may receive a greater blood supply than smaller, more isolated islets, do they have a high/low proportion of beta cells than predicted ? 2) Although we captured some local inflammatory processes in the peri-pancreatic adipose tissue, how these inflammatory processes are distributed along the organ remains to be determined. For instance, are these inflammatory processes confined to the tail region, or do they occur/extend in the head and neck as well ? With regard to the islet clusters one of the methods that appears to be suitable for such an experimental estimate would be the near infrared optical projection tomography for assessments of β-cell mass distribution^44^. Nevertheless, in this study our main observation revealed that islets are spread gradually from the head up to the tail section of the pancreas in the form of contracted or dilated islet routes. If these particular observations can be extrapolated as a general rule in our species, it remains to be thoroughly evaluated in the future.

## Methods

In the present study we have used a healthy human pancreas from a 35 years white Caucasian male donor with a body mass of 75 kg (165 pounds) and a height of 1.82 m (5 feet and 11.65 inches). All experiments were performed in accordance with relevant guidelines and regulations. The methods were carried out in accordance with the approved guidelines. All experimental protocols were approved by the National Organ Donor Network at the request of the Ethical Committee of “NC Paulescu” Institute of Diabetes. Initially, after the removal of the pancreas, both the volume and the length of parenchyma were measured. The immediate step consisted in the histological processing of the pancreas.

### Fixation and processing of the samples

Within a 30 minute interval after excision, the pancreas was immersed in buffered 10% formaldehyde for 24 hours. The fixated pancreas was transversely cut in 4 segments of approximately 6.2 cm, corresponding to the head, neck (isthmus), body and the tail of the pancreas. Next, one transversal slice of 5 mm has been cut from the middle area of each segment and labeled as B1 to B4. Each slice was further divided into a total of 16 samples, labeled as P1 to P16 (Fig. 1A). The samples were fixed in buffered 10% formaldehyde for 24 hours, followed by dehydration in: 1) 70% alcohol for 60 min, 2) 96% alcohol for 45 min, 3) absolute alcohol for 2 h. The clearing phase of the samples was made by repeated xylene immersions, followed by paraffin wax infiltrations. The samples were automatically processed with tissue processor Thermo Scientific STP 120-3 and paraffin embedding was done with modular tissue embedding center Thermo Scientific Microm EC 350-1. Next, the resulting blocks were cut at 5 μm using the Leica RM 125RTS microtome and then carefully placed on the microscope slides. In order to distinguish between tissue types the sections were stained with hematoxylin and eosin by using the Thermo Scientific Microm HMS 70 slide stainer. The histological slides were labeled in the same manner as the samples from which they originated (Fig. 1A). Thus, we established a system of reference by denoting specific areas of slices (ie. B1[P1] - the first histological slide from the head section). The Bx[P1–P4] slides correspond to the dorsal pancreas whereas Bx[P13–P16] slides correspond to the ventral pancreas.

### Microscopic examination and morphometric measurements

The examination of the sections was made with an Olympus BX 41 microscope coupled to an Olympus DP25 video camera. The area and perimeter of the pancreatic islets have been measured by using the Olympus Cell^B analysis system.

### Data analysis

Using classical geometry formulas we have determined a series of parameters, such as the average value of the islet diameter, the mean islet volume, the total number of islets inside the pancreas, the total islet volume inside the pancreas, the total islet 3D surface area and the islet percentage from the total volume of the pancreas (Table 7). The approximation of the total number of islets and the volume that they occupy, has been made separately for each region of the pancreas. Initially, the length of the pancreas (*L* = 250,000 μm) was divided into four (*n* = *4*) regions of equal length (*L/n* = 62,500 μm), representative of the four areas of the pancreas (head, neck, body and tail). Each region was further divided by the average islet diameter (*d*_*i*_). Thus, the new values have been multiplied by the total number of islets (*T*_*i*_). The sum of the resulting values was the representative approximation for the total number of islets (*I*_*tot*_) in the pancreas (7). Likewise, the total islet volume (*V*_*tot*_) has been approximated by multiplying the total number of islets (*I*_*tot*_) to the average islet volume of each region (8). A more direct but less accurate approximation can be performed by multiplying the total number of islets (*I*_*tot*_) to the mean islet volume (*V*_*p*_). In order to determine the islets shapes throughout the pancreas we have devised an index of sphericity which is a measure of deviation from the ideal form of a sphere (9). Heat maps were obtained using several steps. An ellipse shape, meant to represent the pancreas section, was divided into 16 regions in the form of a 4 × 4 matrix (Fig. 1A). A color has been associated to each region based on the number of islets recorded on the corresponding slide. The densest regions have a yellow gradient designation and the least dense regions have a blue gradient designation (method similar to the Nearest-neighbor Interpolation). Depending on the case, other gradient colors were also used. The intensity of each color reflects the number of islets found on that particular region (or the surface area values - depending on the case). In order to approximate the relationship between slide boundaries a Gaussian smoothing function has been applied to the original distribution (10). Thus, *x* represents the distance from the origin in the horizontal axis and *y* represents the distance from the origin in the vertical axis, whereas *σ* is the standard deviation of the Gaussian distribution. Depending on the case, variations of the Gaussian smoothing function have been used. The resulting heat maps were also confirmed through the Bicubic Interpolation method (Supplementary Figure S4 online). The 3D rotational landscapes of the islets have been obtained by using the original heat maps as two dimensional arrays. Thus, for plotting a three-dimensional islet landscape, the x-axis and z-axis correspond to the height and width of the two dimensional array, while the y-axis corresponds to the absolute value of each element of the two dimensional array. The k-means clustering method has been used to establish the 3D routes of islets (14). Where ||*x*_*i*_*−v*_*j*_|| represents the Euclidean distance between *x*_*i*_ (the set of data points) and *v*_*j*_ (the cluster center), *c*_*i*_ stands as the number of data points in *i*^*th*^ cluster and *c* represents the number of cluster centers. The maximum islet density peaks of each slice have been the base line for the connections between routes (*V*) in the metric space. Thus, the islet density peaks of neighboring slices have been used as data points for the k-means clustering method.

## Additional Information

**How to cite this article**: Ionescu-Tirgoviste, C. *et al.* A 3D map of the islet routes throughout the healthy human pancreas. *Sci. Rep.* **5**, 14634; doi: 10.1038/srep14634 (2015).

## Supplementary Material

Supplementary Information

## Figures and Tables

**Table 1:** The number of pancreatic islets found in each histological slide/pancreas section.

Microscope slide	Number of islets found/slide				*Total islets*	*Max*	*Min*
	Head (B1)	Neck (B2)	Body (B3)	Tail (B4)			
P1	44	105	122	0	271	122	0
P2	77	18	129	11	235	129	11
P3	17	17	137	142	313	142	17
P4	77	3	158	215	453	215	**MIN 3**
P5	22	30	233	70	355	**MAX 233**	22
P6	120	32	96	35	283	120	32
P7	44	93	100	113	350	113	44
P8	109	30	179	89	407	179	30
P9	74	27	80	134	315	134	27
P10	13	65	143	94	315	143	13
P11	27	67	95	136	325	136	27
P12	30	39	3	84	**MIN 156**	84	3
P13	100	30	122	98	350	122	30
P14	81	68	89	166	404	166	68
P15	10	41	231	191	**MAX 473**	231	10
P16	65	148	140	65	418	148	65
*Total*	**910**	**813**	**2057**	**1643**	**5423**		
*Max*	120	148	233	215	**473**		
*Min*	10	3	3	0	**156**		
*SD*	±35.77	±38.19	±56.57	±60.45	±81.91		

Bold-Underline = total values or maximum/minimum values specified in the main text.

**Table 2:** The total area (mm^2^) of the pancreatic islets in each section (B1–B4).

Microscope slide	Area/slide (mm^2^)				Total area (mm^2^)	Average (mm^2^)	SD
	Head (B1)	Neck (B2)	Body (B3)	Tail (B4)			
P1	1.821	0.705	0.526	0.000	3.052	0.763	±0.77
P2	0.824	0.192	0.902	0.188	2.105	0.526	±0.39
P3	0.122	0.114	0.614	0.724	1.574	0.393	±0.32
P4	0.877	0.001	1.075	1.475	3.428	0.857	±0.62
P5	0.325	0.274	1.541	0.480	2.620	0.655	±0.60
P6	1.070	0.281	0.673	0.434	2.459	0.615	±0.34
P7	0.190	0.552	0.896	0.990	2.629	0.657	±0.36
P8	1.010	0.255	1.420	0.622	3.307	0.827	±0.50
P9	0.685	0.202	0.747	1.291	2.925	0.731	±0.45
P10	0.130	0.712	0.890	0.503	2.236	0.559	±0.33
P11	0.151	0.546	0.859	0.896	2.452	0.613	±0.35
P12	0.345	0.410	0.041	0.532	1.328	0.332	±0.21
P13	0.647	0.132	0.962	1.275	3.015	0.754	±0.49
P14	0.549	1.259	0.525	2.149	4.481	1.120	±0.77
P15	0.073	0.221	1.532	2.142	3.968	0.992	±1.01
P16	0.432	1.106	0.798	0.487	2.823	0.706	±0.31
*Total*	**9.252**	**6.960**	**14.004**	**14.186**	**44.4**	**0.694**	
*Mean*	0.578	0.435	0.875	0.887	2.775		
*SD*	±0.467	±0.359	±0.391	±0.633	±0.805	±0.201	

Bold-Underline = total or average values specified in the main text.

**Table 3:** Absolute and relative values of the main islet parameters by pancreas region.

Parameters		Head (B1)	Neck (B2)	Body (B3)	Tail (B4)	Mean	SD	Total
Number of islets		910	813	2057	1643	5423	±2026.4	
Sum of all islets	Area (mm^2^)	9.25	6.96	14	14.19	11.1	±3.3	
	Perimeter (mm)	355.32	300.07	653.71	580.85	472.49	±153.7	
Average of all islets	Area (um^2^)	10,155.3	8,561.2	6,807.9	8,633.9	8,539.6	±886	
	Perimeter (um)	390.03	369.1	317.8	353.53	357.615	±22.1	
d_ip_ - Mean Islet diameter (um)		124.1	117.5	101.1	112.5	113.8	±7	
d_ia_ - Mean Islet diameter (um)		113.7	104.4	93.1	104.8	104	±5.6	
d_m_ - Mean Islet diameter(um)		118.9	110.9	97.1	108.7	108.9	±6.2	
r - Mean Islet radius (um)		59.5	55.5	48.6	54.3	54.5	±3.1	
V_p_ - Mean Islet volume (um^3^)		880,805	715,056	479,808	672,306	686,994	±107,297	
V_p_ - Mean Islet volume (ul)		0.00088	0.00072	0.00048	0.00067	0.00069	±0.0001	
A_im_ - Mean islet 3D surface area (um^2^)		44,436	38,670	29,638	37,113	37,464	±4,109	
Pancreas length (um)/islet diameter (um)		2,102	2,253.3	2,573.8	2,300.1	2,307.3	±145.4	
Number of slices/mean islet diameter		23.7	22.1	19.4	21.7	21.7	±1.2	
I_tot_ - Number of islets in the pancreas		478,219	457,990	1,323,603	944,774	801,147	±358,364	**3,204,588**
V_tot_ - Total islet volume (cm^3^)		0.421	0.327	0.635	0.635	0.505	±0.146	**2 cm**^**3**^
V_tot_ - Total islet volume (ul)		421.2	327.4	635	635.1	504.7	±145.6	202
Total islet 3D surface area (cm^2^)		212.5	177.1	392.3	350.6	283.1	±93.9	**1132 cm**^**2**^
Islet procentage from the pancreas volume		0.94	0.73	1.41	1.41	1.12	±0.32	**4.49%**

Bold-Underline = global values specified in the main text.

**Table 4:** Categorisation of the pancreatic islet by their surface area.

Section	Islets by size				*Total*	*Mean*	*SD*
	<= 1000 μm^2^	>1000 and <10,000 μm^2^	>10,000 and <100,000 μm^2^	>100,000 μm^2^			
Head (B1)	37	605	262	6	910	227.5	±276.3
Neck (B2)	57	555	198	3	813	203.2	±248.4
Body (B3)	236	1404	417	0	2057	514.2	±617.2
Tail (B4)	163	1050	428	2	1643	410.7	±460.9
*Total*	493	3614	1305	11	5423	1355.7	±1597.3
*Mean*	123.2	903.5	326.2	2.7			
*SD*	±93.3	±401	±114.2	±2.5			

**Table 5:** The evaluation of islet sphericity.

**Index of sphericity**				
Parameters	Head (B1)	Neck (B2)	Body (B3)	Tail (B4)
*Mean Islet diameter (um)*	118.931	110.947	97.131	108.69
*Average perimeter (um)*	390.03	369.1	317.8	353.53
*I*_*π*_	3.2794	3.3268	3.2718	3.2526
*π*	3.1415	3.1415	3.1415	3.1415
*I*_*s*_	**1.0439**	**1.0589**	**1.0414**	**1.0353**

Bold = global values specified in the main text.

**Table 6:** Estimation of the proportion of occupancy, the total volume and total mass of β cells, α cells and δ cells.

	β-cells	α-cells	δ-cells	Total
*Brissova et al.*^11^	54%	35%	11%	100%
*Cabrera et al.*^9^	60%	30%	10%	100%
*Ichii et al.*^39^	57.4%	32.8%	9.8%	100%
*Mean proportion inside the islet*	**57.13 **± 3%	**32.6 **± 2.5%	**10.27** ± 0.64%	100%
*Volume (cm*^3^)	1.15 cm^3^	0.66 cm^3^	0.21 cm^3^	2.02 cm^3^
*Total mass (grams)*	**1.25** **g**	**0.71** **g**	**0.22** **g**	**2.19** **g**
*% from the pancreas volume*	**2.563%**	**1.463%**	**0.461%**	**4.487%**

Bold = cell type mean values specified in the main text.

Bold-Underline = global mean values specified in the main text.

**Table 7:** Algorithms and mathematical equations used in data analysis.

Description	Formula	Reference number
Diameter of islets using the perimeter	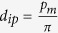	(1)
Diameter of islets using the area	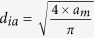	(2)
Mean diameter	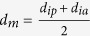	(3)
Mean islet radius	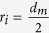	(4)
Mean islet volume	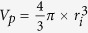	(5)
Mean islet 3D surface area	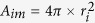	(6)
Total number of islets in the pancreas	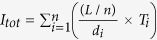	(7)
Total islet volume	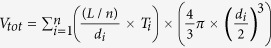	(8)
π	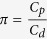	(9)
Ratio between islet perimeter and mean islet diameter	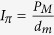	(10)
Index of sphericity	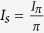	(11)
Gaussian smoothing function	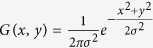	(12)
Total mass calculated according to specific tissue density and islet volume	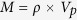	(13)
k-means clustering	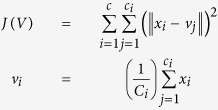	(14)
