# Supplementary material for: A 3D map of the islet routes throughout the healthy human pancreas
Source: Sci Rep. 2015 Sep 29;5:14634. doi: 10.1038/srep14634 (PMC4586491; doi:10.1038/srep14634)
Supplement: Supplementary Information [file srep14634-s1.doc]

**A 3D map of the islet routes throughout the healthy human pancreas**

Constantin IONESCU-TIRGOVISTE, Paul A. GAGNIUC, Elvira GUBCEAC, Liliana MARDARE, Irinel POPESCU, Simona DIMA, Manuella MILITARU

**Supplementary Table S1**. The total area (mm²) and perimeter (mm) of the pancreatic islets in each section (B1 – B4)/slide.

| **Microscope slide** | **Area & Perimeter / slide** | | | | | | | |
| --- | --- | --- | --- | --- | --- | --- | --- | --- |
|  | **Head (B1)** | | **Neck (B2)** | | **Body (B3)** | | **Tail (B4)** | |
|  | **Area (mm^2^)** | **Perimeter (mm)** | **Area (mm^2^)** | **Perimeter (mm)** | **Area (mm^2^)** | **Perimeter (mm)** | **Area (mm^2^)** | **Perimeter (mm)** |
| **P1** | 1.82 | 29.18 | 0.71 | 36.58 | 0.53 | 32.20 | 0.00 | 0.00 |
| **P2** | 0.82 | 30.15 | 0.19 | 7.39 | 0.90 | 42.24 | 0.19 | 5.68 |
| **P3** | 0.12 | 6.19 | 0.11 | 5.65 | 0.61 | 33.21 | 0.72 | 40.91 |
| **P4** | 0.88 | 33.83 | 0.00 | 0.22 | 1.08 | 49.97 | 1.47 | 69.46 |
| **P5** | 0.33 | 11.34 | 0.27 | 12.45 | 1.54 | 71.27 | 0.48 | 22.80 |
| **P6** | 1.07 | 46.90 | 0.28 | 13.21 | 0.67 | 31.11 | 0.43 | 15.15 |
| **P7** | 0.19 | 12.95 | 0.55 | 30.72 | 0.90 | 38.07 | 0.99 | 39.20 |
| **P8** | 1.01 | 42.78 | 0.26 | 11.65 | 1.42 | 62.40 | 0.62 | 28.44 |
| **P9** | 0.68 | 29.89 | 0.20 | 9.84 | 0.75 | 29.48 | 1.29 | 50.60 |
| **P10** | 0.13 | 5.21 | 0.71 | 26.72 | 0.89 | 44.25 | 0.50 | 26.11 |
| **P11** | 0.15 | 8.42 | 0.55 | 25.05 | 0.86 | 35.01 | 0.90 | 41.46 |
| **P12** | 0.35 | 13.47 | 0.41 | 17.02 | 0.04 | 1.46 | 0.53 | 25.88 |
| **P13** | 0.65 | 32.51 | 0.13 | 7.76 | 0.96 | 42.76 | 1.27 | 43.80 |
| **P14** | 0.55 | 27.62 | 1.26 | 37.48 | 0.52 | 26.85 | 2.15 | 70.07 |
| **P15** | 0.07 | 3.18 | 0.22 | 11.87 | 1.53 | 71.58 | 2.14 | 79.05 |
| **P16** | 0.43 | 21.69 | 1.11 | 46.44 | 0.80 | 41.85 | 0.49 | 22.24 |
| **Total area (mm^2^/mm)** | **9.25** | **355.32** | **6.96** | **300.07** | **14.00** | **653.71** | **14.19** | **580.85** |
| **Total area (cm^2^/cm)** | 0.093 | 35.532 | 0.070 | 30.007 | 0.140 | 65.371 | 0.142 | 58.085 |
| **Mean area (mm^2^/mm)** | 0.578 | 22.208 | 0.435 | 18.755 | 0.875 | 40.857 | 0.887 | 36.303 |
| **SD** | ±0.47 | ±13.79 | ±0.36 | ±13.41 | ±0.39 | ±17.48 | ±0.63 | ±22.72 |

Bold-Underline = total area or perimeter of islets.

**Supplementary Table S2**. Comparison between mean area and the number of islets for each microscope slide.

| **Microscope slide** | **Area & no. of islets/slide** | | | | | | | |
| --- | --- | --- | --- | --- | --- | --- | --- | --- |
|  | **Head (B1)** | | **Neck (B2)** | | **Body (B3)** | | **Tail (B4)** | |
|  | **Area (mm^2^)** | **No islets** | **Area (mm^2^)** | **No islets** | **Area (mm^2^)** | **No islets** | **Area (mm^2^)** | **No islets** |
| **P1** | **MAX 1.821** | 44 | 0.705 | 105 | 0.526 | 122 | 0.000 | 0 |
| **P2** | 0.824 | 77 | 0.192 | 18 | 0.902 | 129 | **MIN 0.188** | **MIN 11** |
| **P3** | 0.122 | 17 | 0.114 | 17 | 0.614 | 137 | 0.724 | 142 |
| **P4** | 0.877 | 77 | **MIN 0.001** | **MIN 3** | 1.075 | 158 | 1.475 | **MAX 215** |
| **P5** | 0.325 | 22 | 0.274 | 30 | **MAX 1.541** | **MAX 233** | 0.480 | 70 |
| **P6** | 1.070 | **MAX 120** | 0.281 | 32 | 0.673 | 96 | 0.434 | 35 |
| **P7** | 0.190 | 44 | 0.552 | 93 | 0.896 | 100 | 0.990 | 113 |
| **P8** | 1.010 | 109 | 0.255 | 30 | 1.420 | 179 | 0.622 | 89 |
| **P9** | 0.685 | 74 | 0.202 | 27 | 0.747 | 80 | 1.291 | 134 |
| **P10** | 0.130 | 13 | 0.712 | 65 | 0.890 | 143 | 0.503 | 94 |
| **P11** | 0.151 | 27 | 0.546 | 67 | 0.859 | 95 | 0.896 | 136 |
| **P12** | 0.345 | 30 | 0.410 | 39 | **MIN 0.041** | **MIN 3** | 0.532 | 84 |
| **P13** | 0.647 | 100 | 0.132 | 30 | 0.962 | 122 | 1.275 | 98 |
| **P14** | 0.549 | 81 | **MAX 1.259** | 68 | 0.525 | 89 | **MAX 2.149** | 166 |
| **P15** | **MIN 0.073** | **MIN 10** | 0.221 | 41 | 1.532 | 231 | 2.142 | 191 |
| **P16** | 0.432 | 65 | 1.106 | **MAX 148** | 0.798 | 140 | 0.487 | 65 |
| ***Mean*** | **0.578** | **56.875** | **0.435** | **50.813** | **0.875** | **128.56** | **0.887** | **102.68** |
| ***SD*** | **±**0.46 | **±**35.76 | **±**0.35 | **±**38.19 | **±**0.39 | **±**56.56 | **±**0.63 | **±**60.45 |

Bold-Underline = total values or maximum/minimum values.


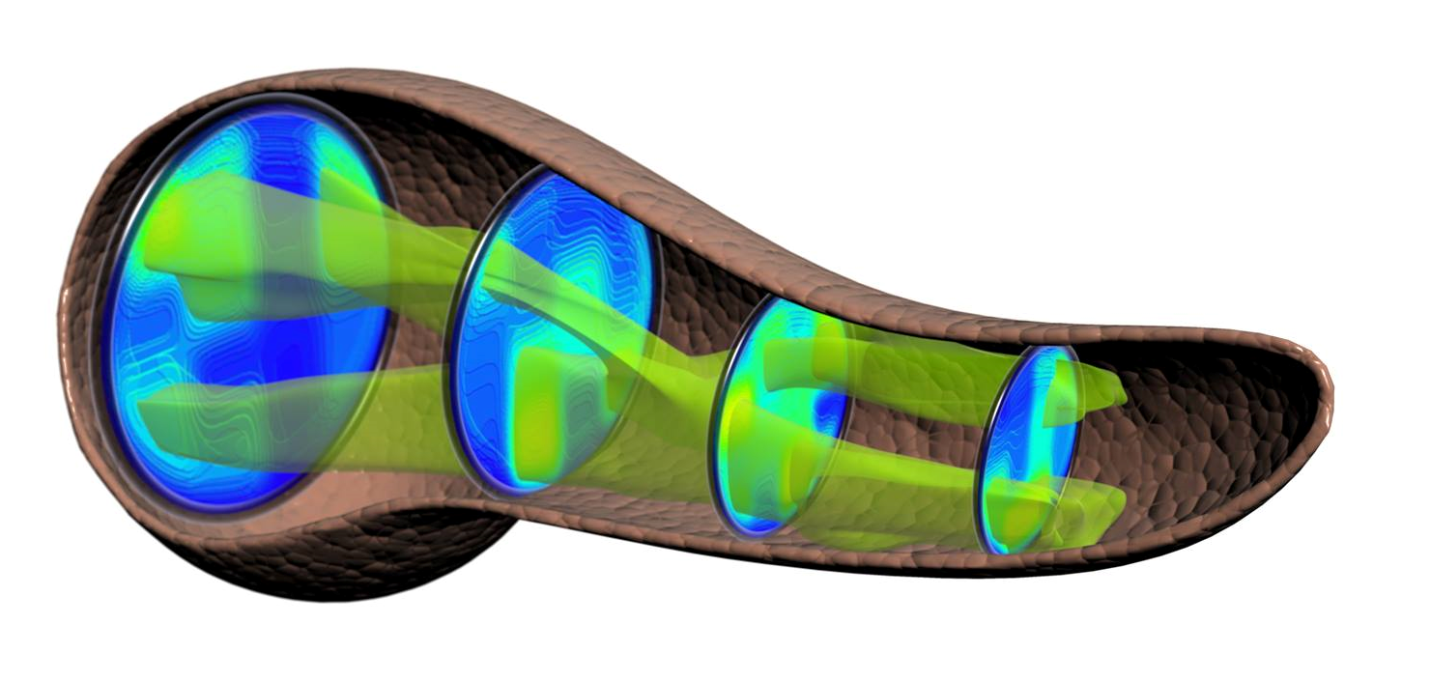


**Supplementary Figure S3.** The 3D representation of the islet routes throughout the human pancreas.


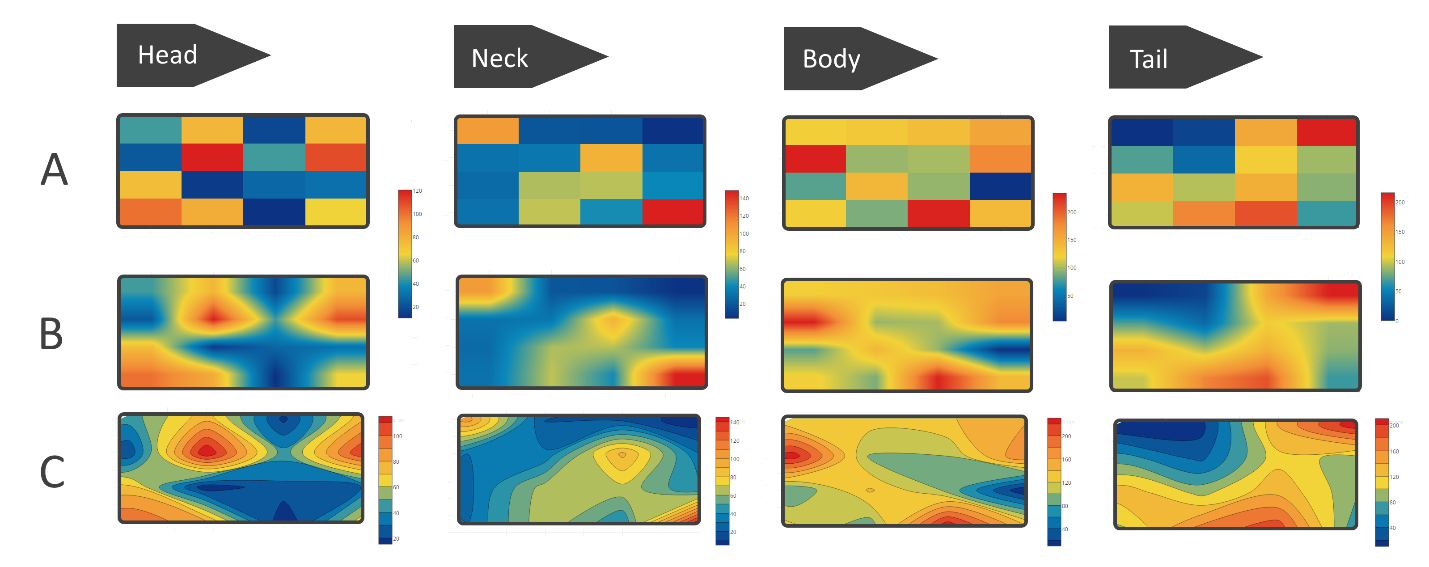


**Supplementary Figure S4.** The 2D distribution of islets throughout the human pancreas. Panels from **A** represent the distribution of islets by Nearest Neighbor interpolation. Panels from **B** represent the distribution of islets by Bilinear interpolation. Panel from **C** represent the distribution of islets through Bicubic interpolation. Slide position is consistent with that described in the methods section of the main article.
